# Immune Checkpoint Inhibitor-Associated Autoimmune Diabetes Mellitus: An Indian Case Series

**DOI:** 10.7759/cureus.97631

**Published:** 2025-11-24

**Authors:** Medha N Rao, Aditi Chopra, Aditya G Hegde, Adarsh K S, Gunna Sriharsha

**Affiliations:** 1 Diabetes and Endocrinology, Manipal Hospital, Bangalore, IND; 2 Diabetes and Endocrinology, Hormone India Diabetes and Endocrine Clinic, Visakhapatnam, IND

**Keywords:** autoimmune diabetes, diabetic ketoacidosis, endocrine emergencies, immune checkpoint inhibitors, immune-related adverse event

## Abstract

Immune checkpoint inhibitors (ICI) have transformed modern oncology but may lead to immune-related adverse events (irAEs), including checkpoint inhibitor-associated autoimmune diabetes mellitus (CIADM), a rare but serious condition marked by abrupt insulin deficiency and frequent diabetic ketoacidosis (DKA). We report five patients who developed CIADM during ICI therapy in India. The patients (three women, two men; mean age: 54 years) received nivolumab or pembrolizumab, with diabetes onset occurring between two and 20 weeks (median: eight weeks) after treatment initiation. Two patients presented with DKA, one with diabetic ketosis, and the others had severe hyperglycemia without ketoacidosis, reflecting heterogeneity in clinical presentation. C-peptide was markedly suppressed in all cases tested, confirming absolute or near-absolute insulin deficiency. Anti-glutamic acid decarboxylase 65 (anti-GAD65) antibodies were positive in three of four patients tested (75%), and antibody positivity correlated with earlier onset and greater metabolic instability. Two patients developed brittle diabetes requiring continuous glucose monitoring and frequent insulin titration. Despite discontinuation of ICI therapy in some cases, insulin dependence persisted, suggesting irreversible β-cell destruction. The timing of CIADM onset in this cohort was earlier than typically reported, highlighting the need for heightened vigilance during the initial treatment cycles. This first Indian case series underscores that CIADM is an uncommon yet clinically significant endocrine irAE. Routine pre-cycle glucose and ketone monitoring during early therapy may facilitate timely detection and prevent life-threatening DKA. Standardised screening strategies, such as baseline and pre-cycle glucose measurement (and ketones, as indicated), as well as streamlined management pathways, are essential to ensure early diagnosis, minimise metabolic complications, and preserve oncologic efficacy without compromising patient safety or quality of life.

## Introduction

Immune checkpoint inhibitors (ICIs), a novel class of immunotherapy agents, have become a cornerstone of modern oncology. This group includes inhibitors targeting cytotoxic T-lymphocyte-associated protein 4 (CTLA-4), programmed cell death protein 1 (PD-1), programmed death-ligand 1 (PD-L1), and lymphocyte activation gene 3 (LAG-3). ICIs may be administered as monotherapy or in combination, depending on the cancer type and clinical context. While these agents have significantly improved outcomes across various malignancies, they are associated with a spectrum of immune-related adverse events (irAEs), particularly those affecting the endocrine system. Among these, checkpoint inhibitor-associated autoimmune diabetes mellitus (CIADM) is a rare but recognized complication, with a reported incidence of 0.087% in a recent meta-analysis of 42,886 patients receiving ICI therapy [1]. Here, we present five cases of CIADM and specifically aim to delineate their clinical presentation, temporal pattern of onset, and metabolic characteristics in Indian patients undergoing ICI therapy.

## Case presentation

Case 1

A 60-year-old man with known type 2 diabetes mellitus (T2DM), well controlled on oral antidiabetic medications (metformin 1,000 mg and vildagliptin 100 mg) and a recent glycated hemoglobin (HbA1c) of 7.4%, was initiated on nivolumab (administered biweekly) for residual esophageal carcinoma.

Following the second dose of nivolumab, he presented with generalized weakness and breathlessness. Evaluation revealed severe hyperglycemia (>600 mg/dL), metabolic acidosis, and positive urine ketones, consistent with diabetic ketoacidosis (DKA). He was managed with intravenous fluids and insulin. Further investigations demonstrated markedly reduced C-peptide levels and a positive anti-glutamic acid decarboxylase-65 (GAD-65) antibody titre, confirming nivolumab-induced autoimmune diabetes mellitus with DKA.

He was discharged on a basal-bolus insulin regimen. Despite discontinuation of nivolumab, he experienced two further admissions for DKA during the subsequent two years, indicating brittle diabetes. At the end of this period, he remains on intensive insulin therapy, and his esophageal carcinoma is in remission.

Case 2

A 59-year-old woman with no prior history of diabetes was diagnosed with stage IV lung adenocarcinoma and initiated on pembrolizumab (every three weeks). Two weeks after the third dose, she presented with vomiting, polyuria, and generalized weakness. Laboratory evaluation revealed marked hyperglycemia (525 mg/dL), positive urine ketones (+++), and an elevated HbA1c of 9.5%, without evidence of metabolic acidosis. Anti-GAD-65 antibodies were positive, confirming an autoimmune etiology. A diagnosis of pembrolizumab-induced autoimmune diabetes mellitus with ketosis was established.

She was stabilized with intravenous fluids and insulin infusion and transitioned to a basal-bolus insulin regimen. Pembrolizumab therapy was initially continued; however, following the ninth dose, she was hospitalized with abdominal pain and diagnosed with pancreatitis, prompting temporary cessation of immunotherapy. Recurrent pancreatic enzyme elevations upon rechallenge led to permanent discontinuation after the tenth dose. Her glycemic control remains unstable, with significant variability on continuous glucose monitoring requiring frequent insulin titration, consistent with brittle diabetes. Her primary tumor remains in remission.

Case 3

A 59-year-old man with no previous history of diabetes was diagnosed with recurrent metastatic renal cell carcinoma and commenced on chemotherapy in combination with nivolumab (every two weeks). After three doses, he developed polyuria, polydipsia, and generalized weakness. Investigations revealed a fasting plasma glucose level of 441 mg/dL and an HbA1c of 8.4%. Urine ketones were negative. He was initiated on a basal-bolus subcutaneous insulin regimen.

C-peptide measured after stabilization was low (0.14 nmol/L), and anti-GAD-65 antibodies were positive, confirming nivolumab-induced autoimmune diabetes. Nivolumab was discontinued after the fourth dose due to disease progression. Unfortunately, the patient died six months after initiation of ICI therapy.

Case 4

A 52-year-old woman with no known history of diabetes was diagnosed with recurrent metastatic squamous cell carcinoma of the gastroesophageal junction. She was started on nivolumab in combination with chemotherapy. Two weeks after the third dose, she presented with abdominal distension, vomiting, and weakness. Laboratory tests revealed severe hyperglycemia (580 mg/dL), positive urine ketones, metabolic acidosis, and an HbA1c of 8.4%. A diagnosis of nivolumab-induced autoimmune diabetes mellitus with ketoacidosis was made.

She was managed with intravenous fluids and insulin infusion and later discharged on a basal-bolus insulin regimen. Nivolumab was continued for an additional 24 doses, during which time she remained insulin dependent. Her malignancy subsequently progressed, and she died the following year due to respiratory failure and sepsis.

Case 5

A 40-year-old woman with hypothyroidism and metastatic carcinoma of Müllerian origin, on nivolumab therapy for five months, presented with generalized weakness and unintentional weight loss. Hyperglycemia was first detected incidentally during an ^18F-D-glucose positron emission tomography (FDG-PET) scan (random plasma glucose of 521 mg/dL). She reported osmotic symptoms, and further evaluation revealed fasting and postprandial plasma glucose levels of 399 mg/dL and 539 mg/dL, respectively, with an HbA1c of 8.6%. There was no evidence of ketosis or metabolic acidosis.

She was initiated on a basal-bolus insulin regimen with rapid symptomatic improvement. Post-stabilization C-peptide levels were markedly reduced (0.04 nmol/L), consistent with insulinopenic diabetes. Anti-GAD antibodies were negative. She remains on basal-bolus insulin therapy and continues maintenance nivolumab.

**Figure 1:**
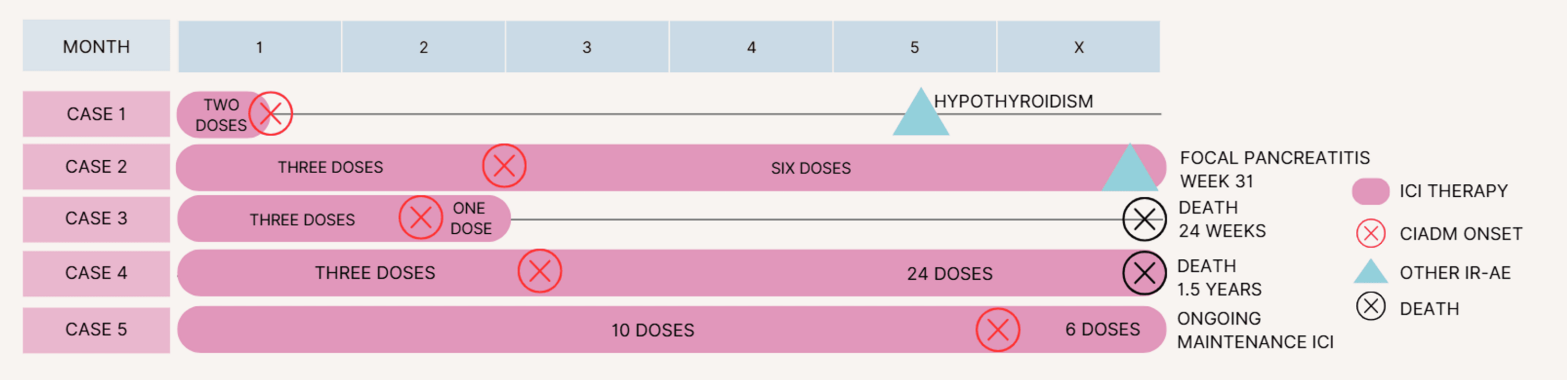
Timeline of therapy and events in the five cases CIADM – Checkpoint inhibitor-associated autoimmune diabetes mellitus; ICI – Immune checkpoint inhibitor; IR-AE – Immune-related adverse event

A detailed summary of the cases is described in provided in Table 1 and Figure 1.

**Table 1:** Summary of cases BMI – Body mass index; C-peptide – Connecting peptide; CIADM – Checkpoint inhibitor–associated diabetes mellitus; CTCAE – Common Terminology Criteria for Adverse Events; DKA – Diabetic ketoacidosis; GAD-65 – Glutamic acid decarboxylase-65 antibody; HbA1c – Glycated hemoglobin; HCO₃⁻ – Bicarbonate; ICI – Immune checkpoint inhibitor; IgG – Immunoglobulin G; irAE – Immune-related adverse event; Nil – None detected; T2DM – Type 2 diabetes mellitus; +/++/+++ – Degree of positivity for urine ketones

Variables	Case 1	Case 2	Case 3	Case 4	Case 5
Demographics					
Age (years)/sex	60/Male	59/Female	59/Male	52/Female	40/Female
BMI (kg/m^2^)	22.2	26.4	23	21.2	22.3
Neoplasia	Esophageal squamous cell carcinoma	Adenocarcinoma lung	Renal cell carcinoma	GE-junction squamous cell carcinoma	Metastatic cancer of Mullerian origin
History of T2DM	Yes	No	No	No	No
Family history of diabetes	Mother	Siblings	No	No	Both parents
History of autoimmune disease	No	Autoimmune hypothyroidism	No	No	No
ICI received	Nivolumab 200 mg	Pembrolizumab 200 mg	Nivolumab 200 mg	Nivolumab 140 mg	Nivolumab140 mg
Chemotherapy received	Paclitaxel, carboplatin	Paclitaxel, carboplatin, fosnetupitant	Lenvatinib	Docetaxel, oxaliplatin, leucovorin	Paclitaxel, carboplatin
Details of diagnosis					
Number of doses before ciadm	Two	Three	Three	Three	Three
Time to onset from the first dose	Two weeks	Eight weeks	Six weeks	Nine weeks	20 weeks
Plasma glucose	> 600 mg/dL	525 mg/dL	441 mg/dL	580 mg/dL	521 mg/dL
Urine ketones	+++	+++	Nil	++	Nil
Metabolic acidosis	Present	Absent	Absent	Present	Absent
PH/HCO_3 _(mmol/L)	7.06/8	-	-	7.12/13.3	-
C-Peptide (nmol/L)	0.02	-	0.14	-	0.05
HBA1C	7.50%	9.50%	8.4%	8.4%	8.6%
Autoantibodies	GAD-65 IgG: >1393 IU/mL	GAD-65 IgG: >2000 IU/mL	GAD-65 IgG: >2000 IU/mL	-	GAD-65 IgG: negative
Pancreatic metastasis	No	No	No	No	No
Presentation type	DKA	Diabetic ketosis	Hyperglycemia with osmotic symptoms	DKA	Hyperglycemia with osmotic symptoms
Other features					
Other ICI-endocrinopathies	Hypothyroidism (TSH 31.9)	Nil	Nil	Nil	Nil
Non-endocrine immune-related toxicities	Nil	Focal pancreatitis	Nil	Nil	Nil
ICI treatment post CIADM	Discontinued	Continued, stopped after pancreatitis	Continued for one dose	Continued	Continued
Tumor response at last follow-up	In remission - 2.5 years	In remission - Two years	Progressed - Death at six months	Progressed - Death at 1.5 years	In remission - Nine months
CTCAE gradings	Four	Three	Three	Three	Two

## Discussion

CIADM is an emerging irAE characterized by rapid and irreversible destruction of pancreatic β-cells, leading to severe hyperglycemia and insulin dependence. Among ICIs, PD-1 and PD-L1 inhibitors, such as pembrolizumab and nivolumab, which were used in our cases, are most frequently implicated, either as monotherapy or in combination with CTLA-4 inhibitors. The proposed mechanism involves disruption of the PD-1/PD-L1 pathway, which normally provides immune tolerance and protects pancreatic β-cells from autoreactive T cells [2].

The first reported case of CIADM appeared in 2012, preceding the U.S. FDA approval of PD-1 inhibitors in 2014. Melanoma and lung cancer are the malignancies most commonly associated with CIADM, likely reflecting the extensive use of ICIs in these cancers [3]. In our series, CIADM developed in patients with gastroesophageal junction carcinoma, lung adenocarcinoma, renal cell carcinoma, and metastatic Müllerian carcinoma, demonstrating that this complication can arise across a wide range of malignancies. To our knowledge, this is the first case series documenting CIADM in an Indian population.

Our series underscores the marked heterogeneity in the clinical presentation of CIADM, ranging from hyperglycemia with osmotic symptoms to frank DKA and, in some cases, progressing to brittle diabetes. The typical age of onset described in the literature is the sixth to seventh decade, which is consistent with the age distribution of most of our patients. While a slight male predominance (~60%) has been reported [2,3], three of our five cases were female. In our series, CIADM onset occurred between two and 20 weeks after ICI initiation, with a median of eight weeks. This median is earlier than that reported in prior studies (12 weeks; IQR: 6-24 weeks) but remains within the broader published range of 1-228 weeks [3,4]. These observations highlight that CIADM may manifest earlier in some patients than commonly described, underscoring the need for close metabolic surveillance during the initial doses.

**Figure 2:**
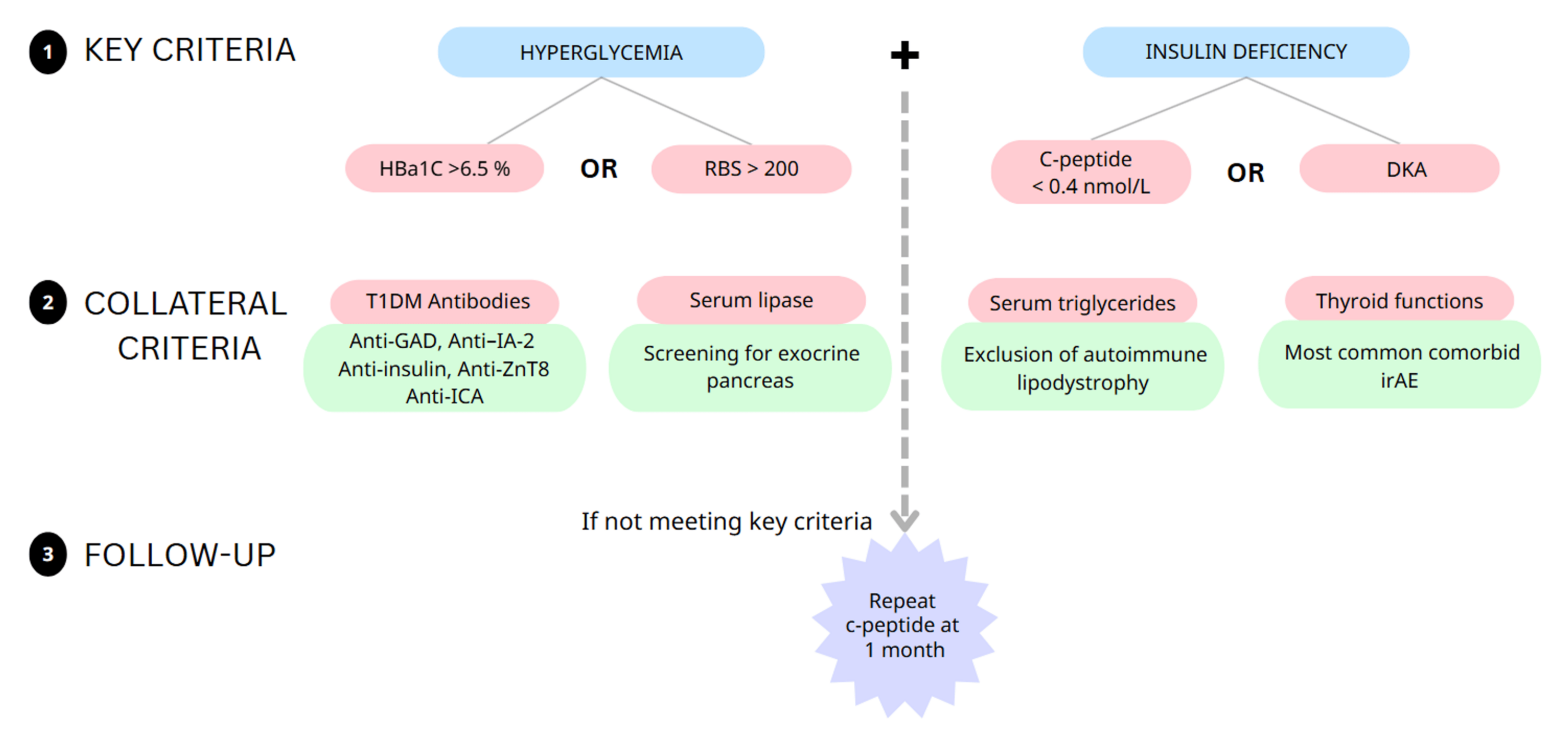
Diagnostic criteria for CIADM CIADM – Checkpoint inhibitor-associated autoimmune diabetes mellitus; DKA – Diabetic ketoacidosis; GAD – Glutamic acid decarboxylase antibody; HbA1c – Glycated hemoglobin; IA-2 – Insulinoma-associated antigen-2 antibody; ICA – Islet cell antibody; RBS – Random blood sugar; ZnT8 – Zinc transporter-8 antibody. Diagnostic criteria for CIADM, adapted from Wu et al. [3].

Diagnosing CIADM can be challenging, often confounded by concurrent chemotherapy, corticosteroid use, and the immunocompromised status of cancer patients. Key diagnostic features include (i) hyperglycemia, defined as a random blood glucose ≥200 mg/dL or HbA1c ≥6.5%; and (ii) evidence of insulin deficiency, indicated by a C-peptide level <0.4 nmol/L and/or presentation with DKA (Figure 2) [3]. All patients in our series met these diagnostic criteria. The average HbA1c at diagnosis in our cohort was 8.5 ± 0.7%, comparable to the reported mean of 8.1 ± 1.5% in larger studies. Among the three patients tested for C-peptide, all had suppressed levels, confirming insulin deficiency. DKA or diabetic ketosis at onset was observed in 60% of our cases, consistent with the 69.7% prevalence reported in the literature [3]. The presence of pancreatic autoantibodies serves as a strong supporting criterion for CIADM. In our series, anti-GAD-65 antibodies were detected in three of four patients tested (75%), which is notably higher than the 40% positivity reported in larger cohorts. Autoantibody positivity in CIADM has been associated with earlier disease onset and a higher likelihood of presenting with DKA [3]. This trend was reflected in our series: the single antibody-negative patient (Case 5) had a relatively delayed onset at 20 weeks and did not present with ketosis. Other supportive diagnostic features include exocrine pancreatic dysfunction and radiological evidence of pancreatic atrophy [3,5,6]. In line with this, one patient in our series (Case 2) developed pancreatitis during the course of therapy.

The diagnostic criteria proposed by Wu et al. [3]; while these criteria are comprehensive, they may lack specificity in patients with pre-existing diabetes, where hyperglycemia and low C-peptide levels may be confounded by the natural progression of long-standing T2DM. To address these limitations, Ma et al. [7] proposed alternative criteria that broaden the diagnostic scope: (i) new-onset fulminant insulin-dependent diabetes or hyperglycemic crisis (e.g., DKA or hyperosmolar hyperglycemic state (HHS)); or (ii) unexplained worsening of pre-existing diabetes or prediabetes, defined as a >50% increase in fasting plasma glucose, the need for treatment escalation (e.g., addition of insulin or another agent), or the development of new-onset DKA, ketonuria, or ketonemia. In our series, one patient with pre-existing T2DM (Case 1) fulfilled these alternative criteria, with additional support from positive anti-GAD-65 antibodies, strengthening the diagnosis of CIADM.

Importantly, all patients had been exposed to glucocorticoids intermittently at some stage of their oncologic management. However, the abrupt onset of metabolic decompensation, close temporal relationship to ICI dosing, and prolonged insulin-requiring hyperglycemia in the presence of low c-peptide or positive antibodies were not in keeping with steroid-induced diabetes and instead aligned with CIADM.

Current screening recommendations advise measuring blood glucose and HbA1c at baseline, followed by pre-cycle glucose monitoring before each ICI infusion for approximately six months, with periodic surveillance thereafter. In the presence of hyperglycemia or suggestive symptoms, further evaluation with ketone testing and acid-base assessment is warranted to rule out DKA [4,8]. Given that the majority of patients in our cohort developed CIADM within the first 12 weeks of initiating ICI therapy, heightened vigilance during this early period may facilitate timely detection of hyperglycemia and help prevent progression to DKA.

All patients in our series were managed with insulin therapy; two developed brittle diabetes requiring continuous glucose monitoring and frequent insulin titration. Acute management of CIADM includes intravenous insulin and fluid resuscitation in cases of DKA or significant metabolic decompensation. High-dose glucocorticoids have no therapeutic role once CIADM is established and may further exacerbate hyperglycemia. In the chronic phase, patients should be transitioned to subcutaneous insulin therapy, typically using a basal-bolus regimen, with close monitoring and individualized dose adjustment. Clinical guidelines, including those from the European Society of Endocrinology, recommend temporarily withholding ICIs during episodes of acute metabolic instability. ICIs may be resumed once adequate glycemic control is achieved on appropriate insulin therapy. Endocrinopathies such as CIADM are not, in themselves, considered indications for permanent discontinuation of immunotherapy [4,8].

In our series, three patients remain alive and on follow-up, while two succumbed to the progression of their underlying malignancy. Unlike pituitary or thyroid irAEs, which have been associated with improved overall survival in some studies, no data currently exist to determine whether CIADM influences long-term survival outcomes [4].

## Conclusions

CIADM is a clinically significant irAE, characterized by abrupt-onset insulin deficiency and persistent insulin dependence in most reported cases. In our series, onset occurred slightly earlier than pooled international data suggest, underscoring that even routine weekly or pre-cycle glucose monitoring during the initial months of ICI therapy may facilitate earlier detection and potentially prevent progression to DKA. A notable finding was the relatively high prevalence of islet autoantibody positivity (75% among those tested), which, although based on small numbers, appeared to correlate with more acute presentation and greater metabolic instability compared with antibody-negative cases. This reinforces the emerging concept that autoantibody status may have prognostic value for disease severity and glycemic trajectory.

Overall, the management of CIADM remains challenging, necessitating an individualised assessment of metabolic stability and oncologic benefit when considering the continuation of ICI therapy. The development of structured monitoring protocols is urgently needed. In practice, this may include baseline HbA1c and glucose, followed by pre-cycle glucose checks (and ketone testing when clinically indicated) during at least the first 12 weeks of therapy, a period when most cases in our cohort manifested. Finally, larger prospective and multicenter studies, including immunogenetic profiling in diverse populations, are needed to better define risk factors, refine diagnostic criteria, and optimize screening and management pathways.
